# Autism: the micro-movement perspective

**DOI:** 10.3389/fnint.2013.00032

**Published:** 2013-07-24

**Authors:** Elizabeth B. Torres, Maria Brincker, Robert W. Isenhower, Polina Yanovich, Kimberly A. Stigler, John I. Nurnberger, Dimitris N. Metaxas, Jorge V. José

**Affiliations:** ^1^Psychology Department, Rutgers Center for Cognitive Science, Center for Computational Biomedicine Imaging and Modeling (Computer Science), Movement Disorders, Neurology, Rutgers University School of Medicine, Rutgers UniversityNew Brunswick, NJ, USA; ^2^Movement Disorders, Neurology Department, Indiana University School of MedicineIndianapolis, IN, USA; ^3^Philosophy Department, University of MassachusettsBoston, MA, USA; ^4^Psychology Department, Rutgers UniversityPiscataway, NJ, USA; ^5^Computer Science Department, Rutgers UniversityPiscataway, NJ, USA; ^6^Department of Psychiatry, Indiana University School of Medicine, Indiana UniversityIndianapolis, IN, USA; ^7^Department of Psychiatry, Christian Sarkine Autism Treatment Center, Riley Hospital for Children, Indiana University School of Medicine, Indiana UniversityIndianapolis, IN, USA; ^8^Computer Science Department, Center for Computational Biomedicine Imaging and Modeling, Rutgers Center for Cognitive Science, Rutgers UniversityPiscataway, NJ, USA; ^9^Physics Department Bloomington, Department of Cellular and Integrative Physiology, Indiana University School of Medicine, Indiana UniversityBloomington, IN, USA

**Keywords:** autism spectrum disorders, stochastic kinesthetic re-afference, Gamma probability distribution, spontaneous behavioral variability, non-stationary statistics

## Abstract

The current assessment of behaviors in the inventories to diagnose autism spectrum disorders (ASD) focus on observation and discrete categorizations. Behaviors require movements, yet measurements of physical movements are seldom included. Their inclusion however, could provide an objective characterization of behavior to help unveil interactions between the peripheral and the central nervous systems (CNSs). Such interactions are critical for the development and maintenance of spontaneous autonomy, self-regulation, and voluntary control. At present, current approaches cannot deal with the heterogeneous, dynamic and stochastic nature of development. Accordingly, they leave no avenues for real time or longitudinal assessments of *change* in a coping system continuously adapting and developing compensatory mechanisms. We offer a new unifying statistical framework to reveal re-afferent kinesthetic features of the *individual* with ASD. The new methodology is based on the non-stationary stochastic patterns of minute fluctuations (micro-movements) inherent to our natural actions. Such patterns of behavioral variability provide re-entrant sensory feedback contributing to the autonomous regulation and coordination of the motor output. From an early age, this feedback supports centrally driven volitional control and fluid, flexible transitions between intentional and spontaneous behaviors. We show that in ASD there is a disruption in the maturation of this form of proprioception. Despite this disturbance, each individual has unique adaptive compensatory capabilities that we can unveil and exploit to evoke faster and more accurate decisions. Measuring the kinesthetic re-afference in tandem with stimuli variations we can detect changes in their micro-movements indicative of a more predictive and reliable kinesthetic percept. Our methods address the heterogeneity of ASD with a personalized approach grounded in the inherent sensory-motor abilities that the individual has already developed.

## Introduction

A core challenge facing research of spectral disorders has been the highly heterogeneous clinical presentation, with manifestation of symptoms varying greatly from individual to individual. In the case of autism spectrum disorders (ASD), individuals show an inherent lack of flexibility, a reliance on sameness, and problems with social interactions. However, even two individuals with the same diagnosis score are rarely alike. The developmental trajectories of ASD can be highly non-linear, ranging from early regression associated with large delays to relatively rapid development associated with advanced skill sets. The adaptive compensatory mechanisms of the autistic individual continuously coping with developmental disturbances are not well-understood.

Current diagnostic practice involves the use of subjective observational inventories (SOIs) based on clinical observations with shifting criteria (e.g., see recent DSM-5 *vs*. DSM-IV-TR debate). Such SOIs provide no objective handle on the heterogeneity of the presentation, and might even obscure individual compensatory capabilities already developed by a coping-adaptive system. In autism the SOI's are primarily rooted in studies involving high functioning boys, with little inclusion of girls, possibly contributing to a steady nearly 5:1 boys-to-girls diagnostic ratio over the years (Volkmar et al., 1993; Lord and Bishop, 2010; Mandy et al., 2012; Dworzynski et al., 2012). Under the current practices many children are missing the optimal window for intervention. There is no way to *objectively* subtype idiosyncratic differences in ASD and/or to dynamically track individual changes in performance *in real time* during behavioral therapies or longitudinally. New methods are also needed to dynamically track the effectiveness of drug therapies on an individual basis.

The SOI's provide criteria for a triad of ASD symptoms that up to now have remained disconnected: (1) problems with social interactions; (2) communication impairment; and (3) repetitive-restrictive behaviors (reliance on sameness). These criteria are based on observation of behaviors. Although behaviors necessarily involve movements, movement disturbances have not been included in the criteria for ASD.

Movements can be performed under voluntary control or occur spontaneously beneath full intentional awareness (Torres, 2011, 2013b). Spontaneous movements and reflexes exist embedded in natural movement sequences and carry rhythms that in typical neonates can be entrained socially e.g., with adult speech (Condon and Sander, 1974) even before perception has fully matured. Retrospective studies of reflexes and spontaneous movements have shown that their disruption precedes the diagnosis of ASD (Teitelbaum et al., 1998; Karmel et al., 2010). On the voluntary side, intentional motions have been documented in neonates as early as 10 days old (van der Meer et al., 1995) continuing along a maturation process that leads to stable goal-directed reaches (Von Hofsten, 1982, 2004; Thelen et al., 1993, 1996; Bhat and Galloway, 2006; Lee et al., 2008; van Wermeskerken et al., 2011). In autism however, typical volitional control is highly compromised often with a striking disconnect between the intentions and the actions of the affected individual (Robledo et al., 2012).

Throughout typical development innate reflexes may initially play a role in the identification of systematic patterns during spontaneous exploratory behaviors by providing reliable referencing anchors. Under typical evolution of reflexes goal-less movements transition into well-coordinated goal-directed acts under volitional control (Thelen and Smith, 1994; Rovee-Collier et al., 2001). In this regard, a hallmark of typical development and maturation is the acquired ability from a young age to flexibly adapt to new contextual situations and interchangeably use and fluidly navigate through spontaneous and intentional patterns of behavioral variability (Torres, 2011, 2013b). This ability might be absent in ASD according to studies of natural motions. We found that the clear distinction quantified in typical controls between goal-directed and spontaneous, goal-less segments of movements was blurred in an individual with ASD (Torres, 2012).

Motor research in ASD has reported life-long persistence of early reflexes, reflexes that typically disappear within weeks of birth (Minderaa et al., 1985; Reed, 2007) as well as other motor disturbances (Damasio and Maurer, 1978; Maurer and Damasio, 1979, 1982; Hill and Leary, 1993; Donnellan and Leary, 1995; Leary and Hoyle, 2009; Donnellan et al., 2013). Yet movement impairments have failed to provide a homogenizing “endo-phenotype” for ASD. Movement disturbances have not been considered a core symptom of ASD and as such are not part of the diagnostic criteria. Perhaps those who diagnose the disorder consider movement disturbances as secondary because of the non-rigorous and subjective ways in which movement has typically been studied in ASD.

Unlike other fields specializing in modeling motion control (Marsden et al., 1989; Doyle et al., 2009) with applications to human behaviors (Todorov, 2005; Bays and Wolpert, 2007; Wolpert, 2007), the ASD sub-field that studies some aspects of motion in human movements has not conceived the stochastic feedback-control nature of motion in biological systems. Along these lines there have been recent attempts to link prior computational models of motor control to autism research (Gowen and Hamilton, 2013). Yet these attempts continue to focus exclusively on intended, goal-directed behavior, consequently disregarding spontaneous behavioral variability and the potential role that it could play in autism. In their present form, computational approaches to motor control cannot address the heterogeneity of the disorder as these models have not been grounded on the empirical estimation of the stochastic signatures of sensory-motor noise/signal of the *individual*. The latter however, are necessary to design personalized therapies tailored to the individual's best abilities.

Here we propose that considering the stochastic nature of both *voluntary* and *spontaneous* motions as separable forms of sensory feedback will shed light on the general question of how we attain spontaneous autonomous control over our actions and make them volitional.

To achieve control and regulation of the motor output in its simplest form, any biological system will require a minimum of afferent sensory feedback in real time. This continuous efferent-afferent flow exchange would enable proper guidance and anticipatory planning of sensory-motor consequences (Kawato and Wolpert, 1998). But besides the goal-driven directionality of the output flow, the temporal transduction and transmission delays inherent to any biological system in the face of sensory-motor noise should also be considered. In the past some of these issues in human motor control have been studied under the general umbrella of internal models (Kawato and Wolpert, 1998; Wolpert et al., 1998) with a focus on goal-directed actions. We posit, however that internal transduction and transmission delays may occur at different time scales for intended and spontaneous motions and that this differentiation, which must be acquired through maturation, may help a system discriminate between levels of intentionality or spontaneity for the same action (Torres, 2013b). Without such separable kinesthetic re-afferent feedback it is hard to understand how a system could turn movement into a tangible percept, fluidly integrate it with other sensory modalities and become cognizant of its own motions, let alone of the motions of others. These ingredients are all crucial for understanding and executing social dynamics in real time. Yet, they have not been considered in movement research in general and in ASD research in particular.

In autism research, movement has been essentially conceived as a form of efferent motor output with a unidirectional flow from the central nervous system (CNS) to the periphery (Jones and Prior, 1985; Rogers et al., 1996; Rinehart et al., 2001; Williams et al., 2001; Noterdaeme et al., 2002; Teitelbaum et al., 2002, 2004; Minshew et al., 2004; Jansiewicz et al., 2006; Mostofsky et al., 2006; Gowen et al., 2008; Fournier et al., 2010a,b) neglecting in more than one way the dynamics of spontaneous behavioral variability patterns inherently present in our motions (Gidley Larson et al., 2008; Haswell et al., 2009; Izawa et al., 2012) and their non-stationary statistics, as pointed out early on by Bernstein (1967).

**Figure 1:**
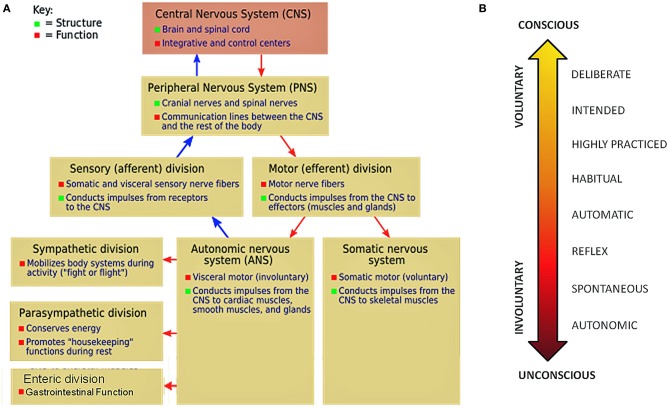
**Levels of the nervous system impacted by sensory-motor noise and gradient of movement variability mapped onto spectrum of movement functionality. (A)** The labels of the simplified schematics represent only some of the many known functionalities of the system. Current autism research primarily focuses on centrally driven goal-oriented tasks typically performed under voluntary control and ignores the dynamics of the peripheral nervous systems (with their autonomic and somatic subdivisions). **(B)** Schematic to show a spectrum of movement functionalities that map onto a gradient of statistical variability. We highlight the need to study movement at all these levels and how autonomic and somatic functions scaffold and contribute to the maintenance of adaptive volitional control and intentional behavior. Both typical development and potential aberrancies are objectively quantifiable in the stochastic rhythms of *all* our motions at accessible levels (e.g., speech gestures, eyes, facial micro-expressions, head, body, limbs, etc.).

To truly understand and appreciate the potential roles that our movements and their inherent variability could play in re-shaping the intentional control of our actions and decisions, we have proposed to treat movements and their variability also as a form of kinesthetic re-afferent input, flowing from the peripheral to the CNSs (Torres, 2013b) (Figure 1A). We have recently introduced the notion that this re-afferent feedback signal gives rise to precise stochastic signatures of movement fluctuations over time (that we have coined “micro-movements”). These micro-movements are proposed to contribute to the regulation, coordination, and control of multiple layers of functionality, in correspondence with a gradient of statistical variability that ranges from autonomic to voluntary levels of control (Torres, 2011) (Figure 1B). At the two extremes of this gradient, behavioral variability from motions voluntarily performed would have different stochastic signatures than behavioral variability from involuntary motions. This is a feature that has enabled blind classification of motion segments of typical subjects (Torres, 2011, 2013b) but failed in a subject with ASD (Torres, 2012).

**Figure 2:**
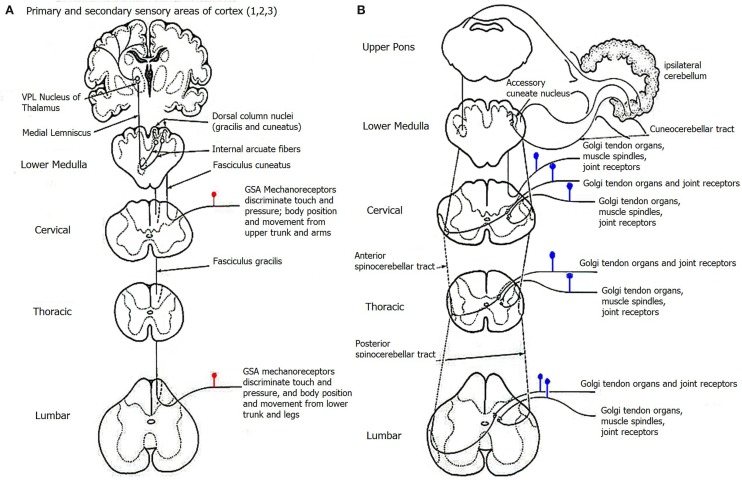
**General Somatic Afferent (GSA) pathways for proprioception. (A)** The so-called conscious proprioception conveying information about touch and pressure and body position and movement through the GSA mechanoreceptors sensitive to discriminating among various levels of the input patterns. Synapses at several points conduct information via the thalamic relay station onto the primary and secondary somatosensory cortices (Broadman areas 1, 2, and 3). **(B)** The so-called unconscious proprioception conveying information about position and movement including dynamics (mass-, forces-, gravity-, and fine internal timing-related information) through Golgi tendon organs, joint receptors, and muscle spindles. Synapses along the way carry information with targets at cerebellar structures.

Parts of the peripheral information involving position, movement, touch, and pressure along with their patterns of variability are routed through general somatic afferent (GSA) fibers: some flow through the so-called “conscious” proprioceptive channels that reach the neocortex via the thalamus, whereas others flow through “unconscious” proprioceptive channels with targets at the cerebellum, striatum, and limbic systems (O'Rahilly and Müller, 1983) (Figure 2). Typically there is balance and flexible exchange between these re-afferent forms of feedback that facilitate central regulation, anticipatory planning, and predictive control of the motor output and its consequences. In autism it is very unlikely that this balance and flexibility remains. Several of the cortical and sub-cortical structures that are targeted by GSA fibers are reported to be impaired along with anomalies involving central and peripheral synapses (Damasio and Maurer, 1978; Maurer and Damasio, 1979, 1982; Jacobson et al., 1988; Rinehart et al., 2002; Amaral and Corbett, 2003; Schumann et al., 2004; Takarae et al., 2007; Amaral et al., 2008; Mostofsky et al., 2009; Qiu et al., 2010; Breece et al., 2012; Nordahl et al., 2012). Problems with the autonomic nervous system (ANS) have also been reported in ASD. These involve the enteric (gastro-intestinal) subsystems (Ashwood et al., 2003; Molloy and Manning-Courtney, 2003; Buie et al., 2010; de Magistris et al., 2010; Kushak et al., 2011; MacFabe et al., 2011; Mazurek et al., 2013) as well as issues with the circadian rhythms (Bourgeron, 2007; Glickman, 2010). Unusual and unpredictable pain and temperature deregulation are well-documented, particularly in autism of known etiology (Nader et al., 2004; Tordjman et al., 2009; Dubois et al., 2010; Klintwall et al., 2011; Zeidan-Chulia et al., 2011; Bandstra et al., 2012).

These disturbances involve motion control at many functional levels of Figure 1B. In ASD such aberrancies are likely to impede *spontaneous autonomy* of the body, body self-awareness, arousal, affective-emotive behaviors, and overall impair volitional control over the person's actions. The above mentioned disturbances are often bundled as “co-morbid” symptoms and downplayed or discarded by contemporary psychological approaches to ASD, despite being widely reported by parents, self-advocates, and other researchers (Donnellan et al., 2013). Proper instrumentation exists to objectively measure many of these disruptions at these various functional levels but adequate statistical methodology has been lacking to tackle these issues in real time and longitudinally in a personalized manner. We show here that the non-stationary stochastic signatures of micro-movements variability and their rates of change in each person can be precisely measured and dynamically tracked over time. They constitute a signature unique to each individual that will help us address the heterogeneity of ASD. They will also help us unveil the best somatosensory-motor capabilities that each person inherently developed along a unique coping and compensatory, adaptive developmental trajectory. We propose ways to use micro-movements' variability as a gateway into the best abilities of each individual with autism.

## Methods

### Participants

We examined a cohort of 78 participants (34 ASD and 44 typically developing TD) ranging from 3.5 to 61 years of age with varying reported IQ. These individuals all were diagnosed as autistic by professionals/agencies qualified to do so and who had no affiliation with our laboratory or this research. Demographic information across participants is listed in Tables A1–A3.

**Figure 3:**
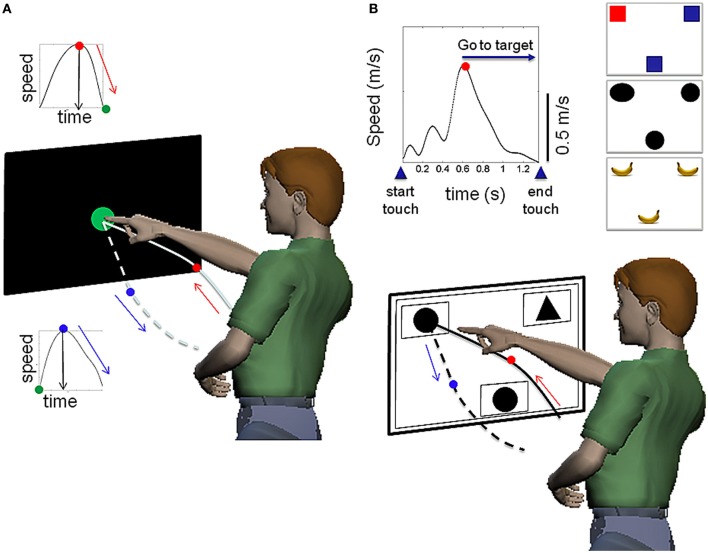
**Two variants of the pointing task to examine goal-directed and incidental goal-less movements in closed loop with decision-making processes. (A)** Variant 1: basic pointing task, measuring the goal-directed motion to touch a target on the touch screen and spontaneous retraction away from it. The arrows mark the flow of motion. The speed profiles are also plotted as insets, with the dot marking the peak velocity and the arrow marking the time at which the peak is attained. **(B)** Variant 2: decision-making pointing during a match to sample task (upper-left and upper-right corners) matches the sample (bottom-center). A representative speed profile is also plotted as insets with the landmarks used to navigate the behavior. The touch at the bottom-center of the touch-screen simultaneously presents the sample and two possible targets. After the decision has been made, the hand goes to the targeted choice and touches the screen again. Examples of other stimuli—of varied cognitive load—used in the match-to-sample task are shown as well.

They performed two versions of a basic pointing task, one which we call “baseline pointing” to a dot. The other one we will refer to as “decision-making pointing” as it is a match to sample task where the target stimuli requiring a decision changes (Figure 3). Reported IQ of individuals with ASD ranged from 40 to 110. For TD individuals IQ is reported 90 and above, with education spanning from pre-school to college levels (22 TD were of college level). The TD children attend the same school as the children with ASD and both are exposed to similar curricular activities. Parents signed parental consent for the children and young adults provided their consent. The protocol was approved by both the Institutional Review Board at Rutgers University and at Indiana University in compliance with the Declaration of Helsinki.

### Task and apparatus

#### Collecting goal-directed vs. goal-less pointing segments

A motion caption system (Polhemus Liberty, 240 Hz) recorded the movements and software [MouseTracker (Freeman and Ambady, 2010)] concurrently time stamped the touches and stimuli presentation, all synchronized to the same CPU. The hand positional trajectories were harnessed. To assess velocity-dependent parameters first-order (velocity) changes in position over time were obtained using the smoothing and derivative functions from the Spline toolbox in MATLAB (MATLAB version 2012a, Natick, MA, The MathWorks Inc.) with software developed in-house. For each velocity trajectory the instantaneous length of the three dimensional velocity vectors along the curve was obtained using the Euclidean norm. A speed profile as a function of time was obtained. In a subset of the participants of college level the MotionMonitor suite from SportsInn, was used to collect data using the Polhemus Liberty (240 Hz) as well. The positional data was filtered using Butterworth filter, 20 Hz cutoff.

**Figure 4:**
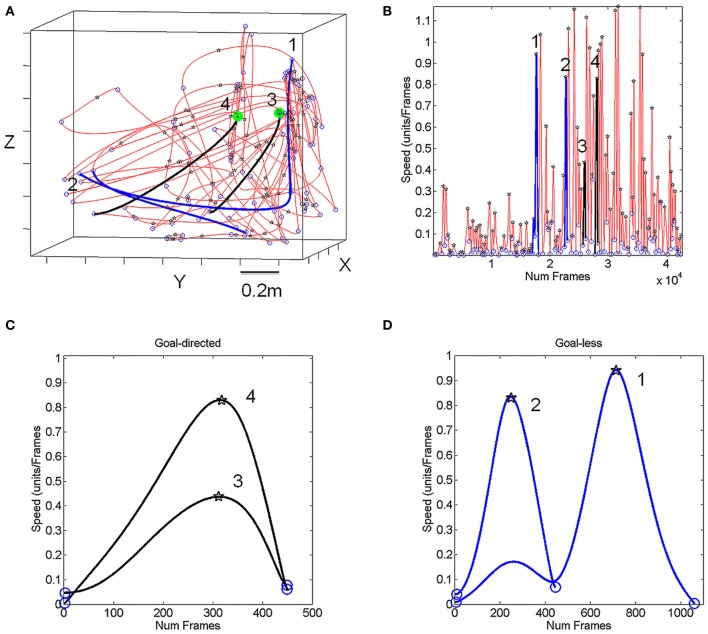
**Navigating through the continuous flow of natural motions and separating goal-directed from goal-less segments of behavior. (A)** Hand movement trajectories from a typical child collected during 16.7 s of the match-to-sample task (at 240 frames/s) which required deciding between two stimulus choices. The blue circles mark the speed minima (pauses) while the black stars mark the speed maxima. The black curves denote the pointing trajectories to the green target at two different positions on the monitor facing the child. The blue curves are incidental to the task, goal-less movements in transition to other goal-directed motions. **(B)** Corresponding speed profiles along the trajectories in **(A)**. Numbers and colors correspond to the curves in **(A)**. The speed temporal profile permits to navigate through the acceleration and deceleration phases of the continuous flow of motion. **(C)** Zooming into the goal-directed speeds and **(D)** the goal-less speed profiles which were automatically harnessed by a computer interface (see methods in the main text).

The baseline pointing paradigm is depicted in schematic form in Figures 3A,B shows the decision-making pointing schematics for the match-to-sample task with representative stimuli types depicted in the top-right corner of panel 3B (e.g., circles, oval, and rotated bananas). Movements were unconstrained in three-dimensional physical space and performed naturally—self-paced and without pre-defined temporal constraints—in a setup similar to that of the children's classroom settings involving a desk and computer screens that the children typically interact with. Figure 4A shows representative hand trajectories from natural motions in one block of trials lasting 16.7 s (40,000 frames recorded at 240 frames per second) including the continuous flow of motion throughout this block. The pointing motion segments had to be extracted from this natural flow and separated from the rest (the incidental transitional segments).

During the experiments the children freely moved and interacted with the touch screen. They triggered the trials by touching the screen which displayed the sample to match. The targets (2 choices) appeared and they chose the target by pointing. The landmarks in Figure 4A are the speed minima (blue circles) and the speed maxima (black stars). The black trajectories are representing pointing movements toward the green target locations (the target on the touch screen). Such movements will be termed “goal-directed” throughout the paper. The blue trajectories are representative of incidental movements that connected the goal-directed ones. These will be termed throughout the paper “goal-less” movement segments. These movements occur spontaneously, largely beneath awareness. Both movement classes were automatically extracted from the continuous flow of the behavioral trajectories by a software interface developed in house.

The Figure 4B shows the speed profiles corresponding to the trajectories in Figure 4A lasting 16.7 s. The blue and black segments correspond to the goal-less and goal-directed segments highlighted in Figure 4A. The panels 4C,D zoom in these sample speed profiles and show the speed minima (blue circles) and maxima (black stars) as those plotted along the trajectories. The numbers identify the segments.

A computer interface logged and time stamped the screen touches to automatically navigate the behavior and separate the goal-directed segments from the goal-less ones. The screen touches were the behavioral landmark delimiting these segments. Backtracking along the valleys and peaks of the hand speed profile from the screen touch to the previous stop of the hand yielded the goal-directed segments. The movements away from the target starting right after the screen touch until the next full stop yielded the goal-less segments. The speed profiles from each movement type were harnessed and examined under a new statistical platform for behavioral analyses (SPBA) (Torres and Jose, 2012).

**Figure 5:**
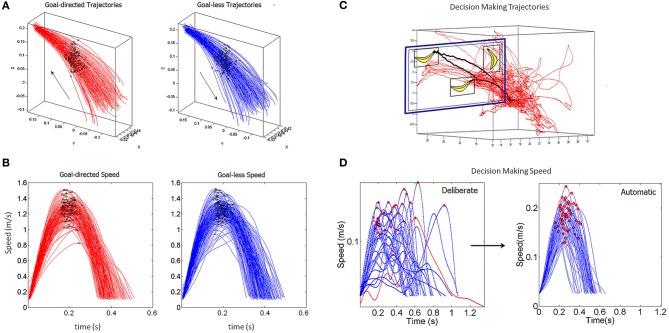
**Sample hand trajectories and speed profiles from the two variants of the pointing task. (A)** Forward movements to the goal and incidental goal-less segments of the basic pointing task performed by an adult participant. The arrow marks the direction of the movement. The stars mark the spatial location of the peak velocity along the trajectories. **(B)** The corresponding instantaneous speed profiles along the trajectories in **(A)**. Notice that these are deceptively similar as the differences lie in their stochastic signatures across trials. **(C)** Decision-making hand trajectories from a child participant. Black trajectories highlight the extraction of goal-directed paths in contrast to goal-less segments. **(D)** Sample speed profiles during the goal-directed decision-making paths. Red dots mark the peak velocity. Red speed is featured in Figure 3B. Notice the evolution from slow and multimodal to fast and unimodal profiles within seconds. The former appear with higher cognitive loads and evolve toward the unimodal “bell-shaped” profiles that are the hallmark of automatic point-to-point behavior in primates.

Sample trajectories from the baseline pointing are shown in Figure 5A for the goal-directed (left) and goal-less (right) segments. In this case (an adult) the movements were more structured than those of the children (e.g., shown in Figure 4). Along the trajectories we also plot the speed maxima corresponding to the single peak in Figure 5B. We are interested in the statistical properties of the spread of the speed maxima and on the spread of the time to reach the maximum speed for both movement types.

The SPBA treats the speed-dependent variations from trial to trial as a stochastic process over time. Specifically we are interested in the *micro-movements* that these parameters describe from one trial to the next. Taken in isolation, these small fluctuations in the value of the movement parameter say nothing about the person's behavior. Yet, over time, they accumulate evidence of the continuous flow of physical behavior, which we can study as a stochastic process. Based on their frequency distributions we can experimentally estimate their probability distributions and examine the evolution of the stochastic signatures in real time as well as longitudinally across different sessions.

This framework does not assume a priori that the data distributes normally (so as to take an average of a given parameter over *n* trials). This assumption is common in ASD motor research, where the theoretical Gaussian distribution is often used to describe the behavioral outcome by the mean and the variance of the parameters of interest and/or perform ANOVA (analyses of variance) and regression analyses on the movement data. Instead, we here experimentally estimate, for each person, the probability distribution most likely describing the movement trajectory parameter. This must be done, as we have previously shown that these velocity-dependent micro-movements do not distribute normally in young healthy adults (Torres, 2011). Normality is a requirement for justifiable use of the mean, variance, and parametric models (Limpert et al., 2001; Limpert and Stahel, 2011), but it has not been properly tested in ASD motor research.

The micro-movements permit proper estimation of the underlying distributions of motor control parameters in a personalized manner and serve to reliably predict different levels of intentionality in the individual's actions (Torres, 2013b). Using the SPBA it is possible to statistically index the predictability and the reliability of the probability distribution estimated from the experimental data as the actions continuously unfold.

To navigate the continuous flow of natural behaviors we had to consider additional issues in pointing during decision-making. The natural trajectories of the hand shown in Figures 4A, 5C contained both multimodal and unimodal profiles (Figure 5D). The latter had smooth slow-down-speed-up sub-segments with no full stops and were associated with exploratory motions as the decision was being made. In such cases the change in the slope of the speed curve was not abrupt—as when the hand comes to a full stop—and above the 5% cutoff from the speed maximum of the segment. Over repetitions of the pointing act, the unimodal speed profiles were re-acquired, indicating that the motions became ballistic and had the signature of automatic reaches. We quantified such adaptive transitions in the speed profiles and in the decision-making parameters. These features enabled automatic segment extraction during decision-making. MATLAB software was developed in-house to detect such subtle differences in densely sampled data.

#### Parameters of interest

***Micro-movement parameters.*** Micro-movement parameters included the maximum value of the speed (*m/s*) and time (*s*) at which these occurred (computed in each trial). The average speed of each trial was also obtained. To remove allometric effects of body-size across ages in each trial we gathered the normalized peak velocity (the peak velocity divided by the sum of the peak velocity and the averaged trial speed) (Mosimann, 1970; Lleonart et al., 2000).

***Decision-making parameters.*** Decision-making parameters included the accuracy of the decision in the match-to-sample task (measured as the % correct) and the movement decision latency (*s*). Movement decision latency was measured as the time (*s*) from the onset of the stimulus (evoked by the participant touching the bottom-center of the screen Figure 3B) to the screen touch at the targeted choice. This includes the reaction time, the time spent deciding, and the actual movement time. Subtracting the movement time (which the speed profile yields between the two relevant minima) provides the decision latency (*s*). Changes in decision accuracy and latency over time were measured in response to different stimuli (Figure 3B) by comparing the first 150 trials to the last 150 trials for each subject. This comparison also enabled us to assess possible fatigue and/or attentional distraction effects. Non-parametric statistics were used to assess significance, as the distributions of these parameters turned out to be highly skewed.

### Distributional analyses

**Figure 6:**
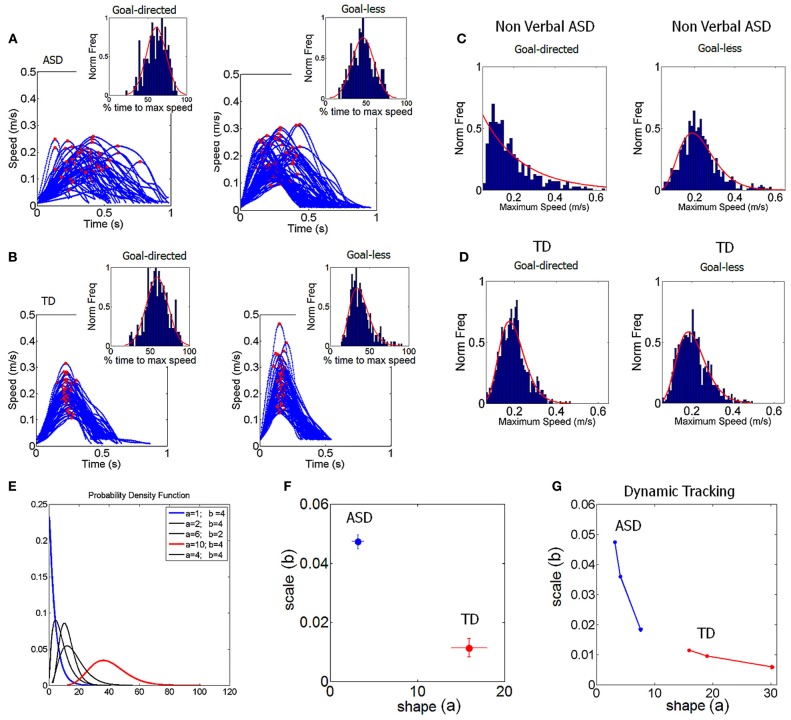
**Simple objective metric to screen idiosyncratic differences and to dynamically track progress in ASD. (A)** Instantaneous speed profiles from a low-functioning non-verbal child with ASD across a subset of trials in one session. Red dots mark the maximum speed value of each trial, goal-directed-forward (left) *vs*. incidental goal-less segments (right). Insets are the normalized frequency distributions of the percent of movement time (s) to reach the maximum speed compiled across sessions. Over thousands of repetitions, this parameter distributes normally in the child with ASD for goal-less reaches. **(B)** Speed profiles of a TD child from a subset of trials in a session (age matched control). The distribution of the percent of time to reach the maximum speed for goal-less segments is skewed. This was the general trend across groups. **(C)** Refers to **(A)**, normalized frequency distribution of the value of the speed maximum compiled across sessions for thousands of trials (goal-directed reaches ASD) well-fit by an Exponential distribution. Retraction segments span an asymmetric distribution. **(D)** Refers to **(B)**, normalized frequency distributions of speed maxima for the goal-directed and goal-less motions of the TD child. **(E)** Schematics of the continuous probability Gamma family with shape (*a*) and scale (*b*) parameters to illustrate that the continuous two parameter Gamma family of probability distributions captures the broad range of cases, spanning from low-functioning, non-verbal ASD to high-functioning, verbal ASD to TD children and young adults (blue curve is the ASD and red the TD cases). **(F)** The MLE of (*a,b*) uniquely localize each child on the Gamma-plane with the 95%-confidence intervals. **(G)** Examples of personalized stochastic trajectories constructed by measuring the stochastic signatures of velocity-dependent hand micro-movements in response to each stimulus type in the match to sample task. Notice that the rate of change of the stochastic trajectory is unique to each child.

These analyses are explained elsewhere (Torres, 2011, 2013a,b). Briefly, we used the continuous two-parameter Gamma family of probability distributions to empirically estimate the probability distribution underlying each person's velocity-dependent micro-movements. Figures 6 A–D provide examples of frequency distributions from the micro-movement parameters of interest from the experimental data of 2 participants, one with ASD and one TD. The two parameters (shape and scale) of the Gamma probability distribution were obtained using maximum likelihood estimation (MLE) with 95% confidence intervals. The shape (*a*) and scale (*b*) parameters can then be plotted in the Gamma plane. They uniquely characterize, with high confidence, the stochastic signatures of the micro-movements as they accumulate evidence across trials on the behavior of each individual under each given condition (Figures 6E,F).

### Dynamically tracking the unique rate of change of the micro-movements' stochastic signatures for each individual

Across different task contexts, we can also track the changes in these stochastic signatures and build a stochastic trajectory in parameter space over time as a function of different stimuli. Each point in the stochastic trajectory is a 2D vector that over time changes direction and magnitude. These rates of change of position in the Gamma plane can also be dynamically tracked in real time and longitudinally. They are unique to each individual. In Figure 6E we show samples of two extreme limits of the Gamma family of probability distribution for two children, one with ASD and one TD. The blue curve (ASD) is an Exponential probability distribution and the red curve (TD) is a skewed distribution tending toward the Gaussian distribution limit. The former describes a totally random process where previous events do not contribute to the prediction of later events, whereas in the latter previous events do contribute to the prediction of future events. The baseline stochastic signatures for these children are shown with confidence intervals in Figure 6F and the stochastic trajectories of each child corresponding to three different stimuli in the match to sample task are shown in Figure 6G.

Lastly there are two important additional methodological steps: we performed (1) a Blind Classification of the cohort, and (2) a Verification step.

Within a cohort, the individuals with similar micro-movement variability will automatically cluster together, as their (*a,b*) stochastic signatures will be close in the Gamma plane. In contrast, those with dissimilarities in the variability of their micro-motions will fall far apart on the Gamma plane. This is an important advantage of this method, as subjects are not grouped a priori (using e.g., K-means algorithm or related clustering methods with preset cluster numbers). Rather it is the inherent statistics of the parameters that determine the groupings (Blind Classification step). Various subjective clinical assessment scores can then be used to find which one best fits within each and across the self-emerging clusters of micro-movement phenotypes. Thus, in assessing ASD the subjectively determined scores and the objective micro-movement metrics can complement each other. Together they would provide an important improvement over the current methods.

Atypical micro-movements might be perceptible to some experienced clinicians (through their own fine-tuned visual perception of movement), but cannot be captured under the current diagnostics categories, which focus on intended and high-level cognitive behaviors. However, under this framework these movements that occur largely beneath awareness can be objectively documented. This is of particular importance in assessing individuals who may not be able to report their self-inferences.

## Results and discussion

This section describes the results from the analyses of hand kinematics with a focus on the velocity-dependent parameters, as well as from the decision-making related parameters of latency and accuracy. The scatter of points obtained as described above in the Gamma plane were colored by age. We used the reported IQ scores in the validation step to obtain a qualitative assessment of the cohort. The blind clustering step produced self-emerging aggregates, which we used to obtain an ensemble plot on the Gamma plane for both the goal-directed and the goal-less segments. An empirical relation between the scale and shape parameters revealed a power-law fit for each case using the expression *f*(*x*) = *mx*^*n*^. We report the exponents (linear regression slope) and goodness of fit of the parameters in Table A3.

### Support for methodological hypotheses

These experimental results that we will describe shortly provide support for our proposed methodological hypotheses (Figure 1) and carry several important specific implications:

1. The trajectories of the stochastic signatures and their rates of change with stimulus type were unique to each person and best described by a range of probability distributions within the Gamma family.
2. Based on inherent similarities in their movement parameters sub-groupings self-aggregated. These were confirmed using the SOI criteria.
3. Given that micro-movements are affected by sensory stimuli, we can drive the system with different forms of sensory guidance. We can then record the motor and cognitive-decision output parameters and readily determine which form of guidance is the most efficient. Efficient here refers to the steering of re-afferent kinesthetic input toward higher predictive and more reliable statistics of the velocity-dependent micro-movements. The latter accompany faster and more accurate decision-making.
4. Since the rate of change of the stochastic signature is unique to each individual and since the variability in goal-directed and in goal-less segments can be studied in tandem with decision-making, we can determine which of these types of processes a person uses most efficiently.
5. This implies that we can very precisely and objectively tailor interventions to each person (even non-verbal participants) and dynamically adapt these new personalized therapies as a function of the inherent capabilities of the person, as their progress unfolds.

### Acquisition of predictive and explorative micro-movements in TD individuals

**Figure 7:**
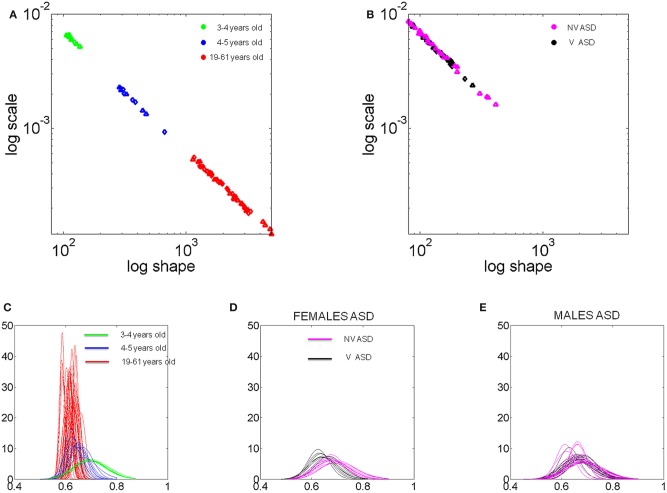
**Typical and atypical development of micro-movement patterns.** The stochastic signatures of velocity-dependent variability captured in the normalized maximum speed (V_max_/(V_max_ + V_avrg_)) of hand pointing motions across different ages. The shape and scale estimated parameters of the continuous two-parameter Gamma probability distribution family uniquely labels each individual in the group (78 participants total). **(A)** Fourty-four typical controls self-cluster by age along the line of unity on the log-log Gamma plane according to a scaling power law. **(B)** Thirty-four participants with ASD also align on the line of unity. Notice that the 34 ASD participants include verbal and non-verbal subjects, spanning from 4 to 25 years of age, yet they all fall along the statistical region of the TD 3–4 years old. **(C)** Estimated probability distributions of the velocity-dependent parameter for all TD subjects using their empirically obtained hand speed profiles. Notice that the noise-to-signal ratio changes dramatically from 3–4 to 4–5 years of age along with the bandwidth of parameter values that the distribution spans across subjects (*p* < 10^−5^). All the 3–4 year old subjects collapsed on the same curve with the noise overpowering the signal but the 4–5 year old subjects have acquired a kinesthetic percept with significantly lower noise-to-signal ratio and broader bandwidth. Such diversification of the kinesthetic input is maximal in the adults who have highly reliable and predictive kinesthetic input. **(D,E)** The kinesthetic input of the ASD participants—unlike that of the 3–4 TD—was unreliable and noisy with narrow bandwidth of parameter values. However, some of the verbal females with ASD separate from the non-verbal females with ASD and from all other subjects (black curves with lower dispersion and centered at 0.62) but do not quite reach the level of kinesthetic input reliability of the TD. The micro-movements in the ASD males are indistinguishable between verbal and non-verbal participants.

We uncovered a scaling power law characterizing the typical maturation process of the stochastic signatures of velocity-dependent micro-movements (Figure 7A). We note the automatic clustering along the line of unity of the (*a,b*) stochastic signatures estimated from the normalized peak velocity. In the bottom panel we show the actual empirically estimated probability distribution for each person. This figure shows the evolution and maturation of the noise-to-signal properties of these distributions. In all participants under 4 years of age the curves showed the highest dispersion according to the Fano Factor (Fano, 1947) [the variance to mean ratio obtained from the (*a,b*) estimated parameters]. Specifically the Gamma statistics revealed significant differences in estimated mean and variance between the self-emerging clusters shown in Figure 7A according to age. Notably the youngest group had the highest dispersion in the probability distribution (*noise to signal ratio*) $F=\frac{\sigma_{w}^{2}}{\mu_{w}}$ taken within the time window between the movement onset and the peak velocity, which was very different between the forward and withdrawing segments (on average 190 ± 50 ms and 70 ± 40 ms, respectively).

In the 3–4 year olds, not only do the movements have a significantly higher variance (leading to a higher noise to signal ratio) than adults (*p* < 10^−5^) but they also operate within a very narrow bandwidth window (low exploration). This implies that regardless of limb size, these young children have unpredictable velocity-dependent variations in their hand movements and do not yet have the systematic diversification necessary for an efficient exploratory trial-and-error learning. This is shown by the broad overlapping green curves in the Figure 7C. Each curve corresponds to a child. Notice that the micro-movements for each child of more than 4 years of age has acquired a broader exploratory range (blue curves) (spanning more values of the mean) for this parameter and the variance (width) significantly decreases. With age the reliability with which the peak velocity can be estimated from trial to trial based on the probability distribution significantly increases (i.e., the Fano Factor decreases). Pair-wise comparisons performing Wilcoxon ranksum test (*p* < 0.0001 comparing children <4 and children >4; *p* < 7.3 × 10^−5^ comparing children <4 and young adults; *p* < 1.9 × 10^−4^ comparing children >4 and young adults).

Across the developmental lifespan these properties and the goodness of fit remained for both goal-directed and goal-less segments. Using the general fitting function *f*(*x*) = *mx*^*n*^ we obtained *m* = 0.77 and *n* = −1.02 with 95% confidence intervals [0.6523, 0.9016] and [−1.058, −0.9918], respectively. The goodness of fit parameters were, Summed-Squared-Error SSE = 5.9 × 10^−8^, *R*^2^ = 0.999, adjusted *R*^2^ = 0.999 and Root Mean Squared Error, RMSE = 6.6 × 10^−5^. Notably the mean value for the Gamma distribution is μ = a*b and the variance is σ^2^ = a*b^2^. Thus, the Fano Factor, *FF* = *b*, which provides the dispersion of the distribution, is also the scale parameter. The higher the value of the scale parameter, the higher the dispersion (i.e., the lower the reliability of the prediction of future events) which is what we see in the youngest children of the group (green dots in Figure 7B and green curves in Figure 7C).

These results suggest that a pivotal maturation in kinesthetic re-afference occurs in TD children around the age of 3–4 years. We consistently found three fundamental developments in the kinematics micro-movements from trial to trial: (1) the value of the shape parameter increases (higher predictability); (2) the noise decreases (higher reliability); (3) the bandwidth of reliable values broadens, thus allowing for efficient exploration. In brief, the development of higher predictive power for future velocities based on past velocities (what is referred to as priors in Bayesian statistics), allows reliable explorative variations: a stable kinesthetic percept is acquired.

We propose that these three factors together make the kinesthetic variations truly perceptual as the predictability along with the reliability of exploratory “sampling” makes it possible through active movements to seek and notice “broken expectations.” They carry information about internal and external environmental constraints. In parallel, decision-making about cognitive stimuli becomes significantly faster (Figure 9F) and more accurate (Figure 10). It is thus no coincidence that TD children universally acquire the “bell-shaped” speed curve around this age (Thelen et al., 1993; Konczak and Dichgans, 1997; Von Hofsten, 2009) which, as we will see shortly, from this age also flexibly re-adapts when faced with new cognitive loads to then return once again to the stable unimodal or “bell-shaped” state. It is important here to note that all children in both groups, TD and ASD, performed this goal-directed task. However, the levels of predictability, reliability, and the bandwidth of their stochastic signatures increased with age, suggesting the above mentioned maturation process.

### Micro-movements go awry in ASD: random, noisy, restrictive kinesthetic input

In drastic contrast to TD development we found that the normalized peak velocity of all 34 participants with ASD across ages and verbal or non-verbal status remained on the region of the Gamma plane corresponding to younger TD (Figure 7B). These included adolescents (14–16 years old) and young adults (18–25 years old). While the noise-to-signal ratio had significantly decreased in the TD 4–5 year olds as compared to that of TD 3–4 years olds (Figure 7A), here there were no significant differences between any of the ASD age-groups (pair-wise comparisons ranksum 4–10 years old *vs*. 16–25 years old test *p* > 0.14), neither between the verbal *vs*. non-verbal types (pair-wise comparisons ranksum test *p* > 0.19). Furthermore, there were significant differences in the noise-to-signal ratios of the participants with ASD and those of the TD participants (rank sum test *p* < 7.2 × 10^−8^).

Besides the noise overpowering the signal in ASD, we also found a lack of diversity in the kinesthetic input. This is appreciated in the Figure 7D where the curves of the Gamma probability distribution of most ASD participants as with the TD 3–4 years old span a very narrow bandwidth of values. Note the contrast to TD 4–5 years old and TD adults who span a large range of values of the mean parameter of the distributions. Thus, whereas the TD cases show a clear transition toward more predictive power, to the right of the Gamma plane, the participants with ASD never transition to lower noise-to-signal ratios and remain with a very narrow range of speed values. The results consistently show that the motions of the participants with ASD do not spontaneously gain the predictability that emerges from and further allows for active autonomous exploration.

This is a crucial finding as all ASD (and no TD) participants showed such unusual normalized peak velocities. It therefore appears to be a unifying characteristic—or endo-phenotype—for the entire autism spectrum irrespective of the heterogeneity of overall clinical presentation. Further, such non-predictability of micro-movements can be hypothesized as directly linked to the pervasive difficulties in ASD with flexibly switching from a set of stable behaviors to another set. We consider this to be one of the most significant and important findings of our studies.

### Velocity-dependent blind clustering and validation of TD vs. ASD participants

**Figure 8:**
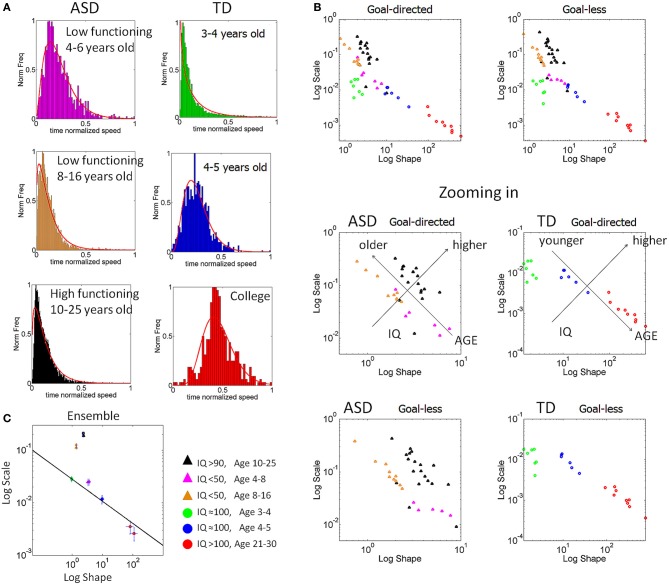
**Self-emerging statistical subtypes for TD and ASD cohorts as a function of age and intellectual abilities. (A)** Normalized frequency distributions from each self-emerging micro-movements' based cluster in **(B)** is shown for the averaged speed in goal-directed segments and incidental goal-less motions. Each distribution is comprised of several thousand trials. Note that the changes in the shape and scale of each frequency histogram are captured well by the continuous Gamma family described in Figure 6E ~15 min into the session upon change in stimulus. **(B)** Scatter of points, where each point represents a participant uniquely labeled by the (*a*-shape, *b*-scale) parameters on the Gamma plane [Table A4 list MLE (*a,b*) values with 95% confidence regions]. Log-log scales are used to depict several orders of magnitude in both axes covering the typical human continuum from pre-school to college. The self-emerging clusters were blindly revealed by the patterns of micro-movements according to the average motion speed. The validation step coloring the scatter according to age and IQ, depicted in the legend, revealed a correspondence with the self-grouping. Bottom panels zoom in the scatters from forward and retraction hand movements. Notice that in ASD the age axis orients the older children away from the typical course of development. **(C)** The stochastic signatures of each cluster are well-characterized by a power relation fit through six points (goal-directed and goal-less for each TD group). The power relation spans several orders of magnitude on both axes (details of the goodness of fit in the main text). Notice again that the young ASD participants fall closer to the TD trajectory in stark contrast to the older verbal and non-verbal ASD participants who stray off the TD path.

Motivated by the results from the normalized peak velocity we assessed the stochastic signatures of the average trial speed. The Figure 8B shows the self-aggregate scatters that automatically emerged according to the micro-movements' fluctuations metric. To gain insights into the clinical nature of each aggregate we colored the dots by age and IQ. This validated the results according to the reported IQ scores in ASD since the orientation of the self-emerging clusters revealed a trend in reported intellectual capabilities (as currently judged by standardized tests) according to verbal skills. This is shown in the zoomed-in panels below Figure 8B.

The coloring gave rise to the empirical frequency histograms in Figure 8A well-fit by estimating the two Gamma parameters in each cluster of Figure 8B. The resulting distribution estimated curves are superimposed in red on the empirical frequency distributions of Figure 8A. Notice that the TD children younger than 4 years old show an Exponential distribution similar to the one observed in the speed maxima for individuals with ASD (Figure 6C goal-directed pointing). This is important, since the Exponential distribution is a memoryless, random distribution, indicating that the fluctuations in the average speed of goal-directed movements are not predictive of the impending speed. Yet in the TD participants older than 4 years of age this statistical feature changes toward a skew distribution so that the kinesthetic percept to which these re-afferent fluctuations give rise becomes more stable (verifiable). By college age the average speed in a past trial does contribute in a predictive manner to indicate future performance according to the more symmetric nature of the frequency distribution of this cluster.

In marked contrast to the young TD 4–5 year old, in the ASD groups older than 8 years old the clusters are closer to Exponential than to Gaussian. See panel 8B on the Gamma plane. The findings thus mirror those regarding the bandwidth of velocity maximum values in TD *vs*. ASD development. However, notice that the non-verbal 4–6 year old ASD group is closer to the 4–5 years old TD group than to the older—both verbal and non-verbal—ASD groups. This is also appreciated in the ensemble data of Figure 8C which is well-fit by a power relation *f*(*x*) = *mx*^*n*^ with *m* = 0.028 and *n* = −0.420, with 95% confidence intervals [0.025, 0.030] and [−0.492, −0.347], respectively. (The goodness of fit parameters were SSE = 4.63 × 10^−6^, *R*^2^ = 0.992, adjusted *R*^2^ = 0.991 and RMSE = 0.0010076). These averaged trial speed results were thus consistent with those from the normalized maximum speed, yet they added more information: (1) the 4–6 year old participants with ASD, were the only ones to approach the area of the TD 4–5 year old, and (2) older individuals with ASD settled into non-predictive and non-exploratory variation patterns further from the TD developmental trajectory than the younger group.

The clusters found in the line-fit (Figure 8C) span several orders of magnitude. They may serve to blindly characterize the pre-school-to-college transition with respect to this metric within a typical developmental trajectory. See further details for each cluster in Table A3. The zoomed-in lower panels of Figure 8B show the suggested orientation axes from the validation procedure. The reported-IQ direction of the blindly determined clusters coincided with the reported clinical scores in both ASD and TD. Validating the axes for the ASD population by age again showed a reversed orientation compared to TD. Notably, even the verbal ASD group veers-off the typical trajectory. We return to this reversed developmental trend in ASD micro-movements in the conclusions, as it highlights the importance of early detection and intervention. It also raises the issue of whether certain symptomatic behaviors in ASD are actually due to active coping as part of an adaptive mechanism in these individuals.

Notice also that the procedure of validating (and coloring) clusters by reported-IQ and age revealed 3 outliers from the verbal ASD cluster. See the zoomed-in lower panel of goal-less segments in Figure 8B. Note that the 2 outliers to the left were the ones scoring highest on the repetitive-stereotypical behavior subscale of the ADOS. Their IQ scores were in the 80–90 range and they have some verbal abilities, yet their somatosensory-motor stochastic signatures placed them in the cluster with the non-verbal individuals with IQs below 50. The third outlier whose signatures fell close to the young TD cluster was recently mainstreamed to a regular kindergarten. Thus, the discovery of those outliers by our approach before knowing their previous test results highlights the individual precision of the micro-movement perspective.

### Decision making-dependent clustering

**Figure 9:**
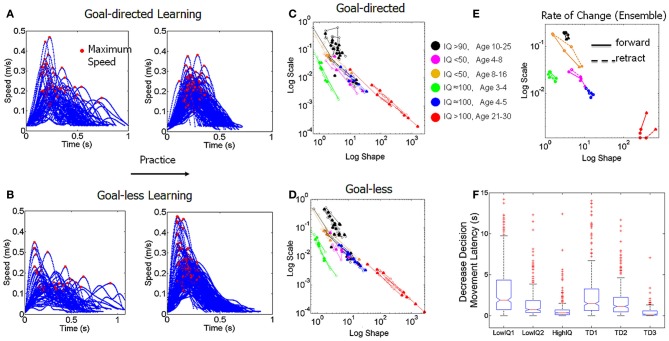
**Dynamically tracking the micro-movements as a function of decision-making. (A)** Speed profiles from a pre-school TD participant showing how changes in cognitive load of the decision-making task initially evoked multiple peaks in the hand velocities to the target, yet unimodality returned within minutes of practice. The stabilization also manifested in the goal-less segments **(B)**. **(C)** log-log plot of trajectories of the rate of change in the stochastic signatures of the average movement speed in the goal-directed hand motions in each self-emergent statistical subtype of Figure 8A (depicted in the legend). Each individual manifested different responses to the change in cognitive load on the micro-movements, and these effects were objectively tracked in each session (open circles represent color and shape, followed by triangle representing rotation). **(D)** The shifts in the stochastic signatures were also tracked in the goal-less non-instructed hand retractions. **(E)** The ensemble data shows greater shifts for the older non-verbal ASD groups. **(F)** With movement practice, the decision-making latencies (shown in seconds) significantly decreased across clusters when comparing the 150 later to the 150 earlier trials of each session (details in main text). Participants with lower IQ and younger TD participants showed the strongest effects. Similar trends on the increase in accuracy are reported in the main text.

The inherent variability in the velocity-dependent parameters from the hand kinematics thus revealed self-emerging clusters which unambiguously separated ASD and TD individuals of comparable chronological ages and IQ. These clusters were then used to assess the decision latency and the accuracy of the decision as the participants performed the match-to-sample task. The decision latency increased significantly according to the Friedman's test when going from color discrimination to discrimination of shapes and rotated objects. Column effects were observed across stimulus type (*p* < 4.9 × 10^−102^, χ^2^ 482.41) and rows effects were observed across cluster type (*p* < 8.5 × 10^−97^, χ^2^ 458.16). The Figure 9A reveals the empirical frequency distributions of this decision-making parameter while Figure 9B shows the localization of the different clusters on the Gamma plane. Here we note that ASD participants above 4 years of age cluster closer to TD participants younger than 4 years than to their same age TD peers. Thus, the cognitive decision latency parameter reveals an atypical developmental trajectory precisely compatible with what we found via the micro-movement parameters. It is important to clarify that the use of term “cognitive” here is reserved for non-motor parameters tied to decision-making. For example, we examine the accuracy and the latency of the decision. This is in contrast to the use of the term cognitive in relation to intellectual capabilities—as we do not know exactly how to measure those in ASD.

Another cognitive parameter impacted by the stimulus change was the accuracy of the decision, which decreased in the non-verbal ASD participants as well as in the young TD participants. The % of errors generally increased from color discrimination to the discrimination of ambiguous and rotated shapes (Kruskal–Wallis *p* < 0.05, χ^2^ 14.99) but with more variability in the color condition errors for the children with ASD and no significant changes for the verbal adults and the college level group (Friedman's test *p* < 0.86, χ^2^ 0.03). See Tables 1–2.

**Table 1:** **Systematic changes in cognitive decision-making performance occurring in parallel with motor (speed) learning**.

	**Color**	**Shape**	**Orientation**
	**Mean (*SD*)**	**Mean (*SD*)**	**Mean (*SD*)**
Percent correct	0.97 (0.18)	0.95 (0.21)	0.91 (0.29)
Decision time (ms)	2285.4 (2370.1)	2570.8 (2926.9)	2739.8 (3527.8)
ASD-DT reduction (ms)	2887 (2168)	1538 (1382)	1344 (843.8)
*P*-value ASD-DT reduction	6.7 × 10^−7^, χ^2^ = 28.41	0.003, χ^2^ = 11.3	0.05, χ^2^ = 5.87
TD-DT reduction (ms)	1556 (1048)	1757 (1480)	2798 (3325)
*P*-value TD-DT reduction	4.5 × 10^−5^, χ^2^ = 20	1.2 × 10^−9^, χ^2^ = 41.02	2.1 × 10^−13^, χ^2^ = 58.37

Percent correct is the total percentage of correct responses for all individuals for each stimulus type. Decision Time (DT) is the length of time from stimulus onset to when the participant touched one of the two targets. DT reduction is the average number of milliseconds by which participants got faster when comparing their performance for the first 50 trials to the last 50 trials for each stimulus type. This gives a measure of performance gains over time. P-values are reported for Kruskal-Wallis comparisons. Number of trials used across stimulus types and subjects: 2546 color; 3588 shape; 2996 rotation. Reductions in latency are reported on the table for the ASD and the TD groups overall. For the individual self-emerging clusters they were significant across conditions for cluster 1 (non-verbal ASD 4–6 years old, p < 0.0002, χ^2^ = 17.97); cluster 2 (non-verbal ASD 8–16 years old, p < 5.8 × 10^−5^); but not significant for cluster 3 (verbal ASD 10–25 years old, p < 0.80, χ^2^ = 0.45). The TD cluster 4 also had a significant reduction in the latency of the decision-making motion (TD 3–4 years old, p < 0.0003 χ^2^ 16.53) but not significant in cluster 5 (TD 4–6 years old, p < 0.49, χ^2^ = 1.42) and in cluster 6 (TD 21–30 years old, p < 0.52, χ^2^ = 1.28).

**Table 2:** **Systematic changes in cognitive decision-making performance occurring in parallel with motor (speed) learning: condition's pair wise comparison**.

	**Color vs. Shape**	**Shape vs. Orient**	**Color vs. Orient**
	**Tukey HSD**	**Tukey HSD**	**Tukey HSD**
Percent correct	0.0001	0.05	0.0001
Decision time (ms)	0.001	0.0001	0.0001

Tukey's HSD post-hoc tests revealed that each group was different from each other group for both Percentage Correct and Decision Time. The direction of significance reveals that Orientation was the most difficult task (fewer correct, longer decision time), whereas color was the easiest task.

### Dynamic, real time tracking of individual adaptive progress

The changes in decision-making stimuli affected hand speed profiles, which gave rise to a re-learning process that we dynamically tracked. As new variants of the task were introduced the hand speed profiles systematically changed from unimodal to multimodal, decreased the accuracy in the children (Kruskal–Wallis *p* < 0.05, χ^2^ 14.99), and increased the latency of their decision-making responses (Friedman test, stimulus effect *p* < 4.9 × 10^−102^, χ^2^ 482.41, cluster effect *p* < 8.5 × 10^−97^, χ^2^ 458.16). Yet within minutes the speed profiles returned to their stable unimodal feature. Thus the introduction of new tasks with different cognitive loads gave rise to a tractable real time learning-adaptation process. This process also revealed that the stochastic signatures of the average hand speed shifted at a different rate, a rate that was unique to each individual in the cohort.

Examples of multimodal speed profiles are shown in the left panels of Figures 9A (goal-directed) and 9B (goal-less). These changes manifested in both TD and ASD groups. After minutes of practice, the speed profiles recovered their unimodality and the movements themselves became faster. This is shown on the right panels of Figures 9A,B. In particular, notice that the time (ms) to reach the maximum speed value was within different time scales in the goal-directed and goal-less motions. The latter had latencies of time to peak velocity on the order of 60–90 ms, which is too fast to reach visual awareness as the hand-eyes are still processing touch-visual information about the chosen target. Statistically significant differences were found across participants in this kinematic latency parameter when comparing goal-directed and goal-less segments (Wilcoxon ranksum test *p* < 10^−6^) whereby in the goal-directed reach the median time to the maximum speed was between 172.95 and 210.53 ms in ASD and between 109.50 and 179.81 ms in TD. In contrast the goal-less segments were between 93.28 and 108.30 ms in ASD and between 60.34 and 152.11 ms in TD. From this result we conjecture that the fast, automated goal-less motions may be routed differently through the sub-cortical “unconscious” proprioceptive GSA fibers (e.g., such as those in Figure 2B.). The forward reaches, where the movement is deliberately launched as a person decides on a matching target show longer latencies to reach the maximum speed. These may be routed through the cortical “conscious” proprioceptive GSA fibers in (Figure 2A).

We also tracked the stochastic signature of each individual by math-to-sample discrimination task: color, geometric shapes, and rotated objects (Figures 9C,D). Here we show the longitudinal trajectories across weeks for the youngest groups (3–16 years old). On the same Gamma plane we show the real time shifts within one session for the older participants (16–30 years of age) from both ASD and TD groups performing the task within one and/or two sessions. Interestingly, some systematically shifted toward the Gaussian range (positive predictive gain), while others moved back (negative random-memoryless gain) or had near-zero gain on the Gamma plane with variable rates that depended on the stimuli. The overall behavior of the ensemble could also be objectively quantified. See Figure 9E for each of the self-emerging clusters. Notice that on this logarithmic scale the older non-verbal ASD cluster showed the largest overall shift toward the typical ranges. Importantly, as the perceptual stimulus changed this was the cluster whose shifts in the stochastic signatures of the goal-less motions maximally differed from the shifts for the goal-directed motions. This distinction became maximal for the decisions on geometric shapes. A possible interpretation of this finding is that this non-verbal ASD group prefers spatial/geometric stimuli in the very precise sense that these allow a better distinction of their goal-directed and goal-less micro-movements than other stimuli. Applied to behavioral training regimens this suggests that these individuals would benefit from usage of geometric type stimuli, which made their motions more predictable and more functionally differentiable in the least amount of time.

Figure 9F shows the decrease in latency (s) for the initiation of the goal-directed movement when comparing the 150 earlier trials to the 150 later trials. The improvements in speed and accuracy of the decision as well as those in the speed of the hand pointing motions render fatigue or attentional effects unlikely in these experiments.

**Figure 10:**
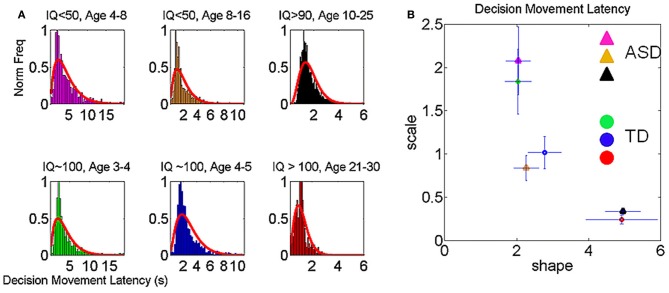
**Decision movement latency across self-emerging clusters. (A)** Frequency histograms of the decision movement time from the self-evoked onset of the sample stimulus to the end of the forward motion at the touch of the screen on the targeted choice. Clusters are from the self-emerging aggregates of the velocity-dependent kinematics parameters. **(B)** Stochastic signatures of this parameter on the (*a,b*) Gamma plane. Notice here that 4–8 year old ASD participants are closer to the 3–4 year old TD participants than to the TD group closer to their own age.

The non-verbal children with ASD and lower reported-IQ experienced the largest improvements in combination with the largest shifts in micro-movements and a different course of performance gains for the goal-less segments incidental to the task (Figure 9E). Table A3 reports further details of this learning progression in the decision-making parameters. Figure 10 shows the distributions of the decision latency using the clusters obtained from the velocity-dependent micro-movements in Figures 7, 8. Thus, through rehearsing, this simple decision-making task, independent of the level of task understanding, reveals statistical shifts toward more predictable micro-movements of their incidental goal-less motions.

Table 1 lists the means and standard deviations for the learning-based reduction in the decision latency across trials and longitudinally for each task. Median values for the reduction in latency per cluster are: TD kindergarten (1601 ms); TD preschool (1203 ms); TD college level (172 ms); non-verbal ASD 4–6 years old (1937 ms); non-verbal ASD 8–16 years old (710.5 ms); verbal ASD 10–25 years old (437 ms). Practice registered significant reductions in the overall movement time for each self-emerging cluster (ranksum test, *p* < 0.05). Along with the reductions in latency, there were corresponding increases in choice-accuracy as measured by the percent correct, *F*_(2, 9130)_ = 52.37, *p* < 0.0001. As indicated by *post-hoc* tests, each group was significantly different from each other group for both parameters. See Table 2 for *P*-values of Tukey's range *post-hoc* tests (Honest Significant Difference tests).

Individuals improved their overall performance longitudinally with practice as evidenced by a significant reduction in the decision time once the speed profile became unimodal and the movement duration was steady (Table 1). As the speed profiles evolved to the unimodal signature of automatic reaches so did their proficiency at the cognitive decision-making task. Both TD and ASD participants showed cognitive effects of having to adjust to new tasks. However, both the older verbal ASD and the college-level TD group adjusted to the change in stimulus faster than the children and their accuracy rate was nearly 100% for each stimulus type (Wilcoxson ranksum test across thousands of trials *p* < 0.46).

These similarities between the adults with ASD and the TD adults are interesting given the findings that the stochastic signatures of their micro-movements were fundamentally different during the decision-making pointing. This suggests that rather different mechanisms were used to attain accuracy in each case. Proprioceptive input was random, (unpredictable), noisy (unreliable), and non-diversified in ASD. They were able to maximally distinguish goal-directed from goal-less motions only in the spatial geometric stimulus set. Given these results, it is possible that they were relying on the actual spatial physical stimulus present throughout the decision. This is in contrast to reliance on an automated, embodied version of it, as we believe was the case for the TD participants. Further testing of this supposition is warranted given also that other researchers have reported that people with ASD rely on visuospatial strengths to perform cognitive tasks that do not, with neurotypicals, require such skills (Samson et al., 2012). In that report Mottron and his team (which, interestingly, includes individuals with autism) have suggested that the over-reliance on complex visualization may be a successful adaptation and, indeed, provide further support for the neurodiversity model of autism.

## Conclusions and future steps

Our work introduces a new unifying statistical framework and a set of objective metrics to tackle the heterogeneity of spectral disorders. This is a personalized approach to the analyses of real time behavior and to assess longitudinal changes in general. Using this approach it is possible to dynamically track not only the natural developmental trajectories of the individual but also the rates of accelerated change as a function of sensory stimuli in general. The new conceptualization of micro-movements not only as efferent signal but also as kinesthetic re-afferent signal is bound to have a broad impact for the study of behavior across various disciplines. From movement neuroscience to sports science to computer science and robotics, this new biologically plausible notion of behavioral variability along with the new statistical platform for dynamic stochastic tracking could transform the ways in which we study and assess learning, adaptation, normal development, and normal aging. The new framework will also enable us to detect atypical patterns and track those patterns as they evolve both in real time and longitudinally.

The micro-movements are here hypothesized to reflect layers of multi-directional internal and external influences on the central and peripheral nervous systems (Figures 1A,B). The re-afferent nature of the velocity-dependent micro-movements paired with their precise measurements at the motor output open the possibility of unveiling potential regulatory control and adaptive mechanisms of the typical system as well as their different manifestations in the specific case of ASD.

This approach can thus inform the search for “endo-phenotypes” across the autism spectrum. Studying physical hand movements allowed us to rapidly detect autistic traits and track the idiosyncratic rates of change in micro-movements *for each individual*. Our methods permitted tackling such issues in real time during decision-pointing actions, as well as longitudinally across different experimental sessions. We found that micro-movements serve as a putative biomarker of typical proprioceptive-motor development in the limbs as well as to flag deviations from the typical developmental path. Applied to 34 individuals with a diagnosis of ASD we blindly detected clusters of individuals with similar micro-movement features and validated that these anomalies in micro-movements corresponded to degrees of verbal capabilities as well as to clinically reported IQ scores.

The classification approach developed here demonstrated that the new framework can address the heterogeneity of the disorder and blindly sub-type autism severity according to the subject's verbal capabilities without a priori choosing the clusters or trying to homogenize the various groups. Our approach also addresses the non-stationary statistical nature of natural behaviors.

An important aspect of the new metrics is that they enable the identification in real time of the type of sensory input which can accelerate learning by steering the person's proprioception toward more predictive behavioral regimes with faster and more accurate decisions. This is because we have precise ways to detect changes in such stochastic patterns toward predictive or toward random regimes. We can assess the statistical reliability (noise-to-signal ratios) in the context of all natural movements—goal-directed segments or goal-less segments occurring beneath awareness. We can selectively find and use the form of sensory-motor guidance that makes the individual more efficient at choosing and controlling adequate motor programs in the face of sensory-motor noise. Micro-movements therefore offer a new way to automatically track improvements, and reinforce the re-afferent sensory-motor input that leads to predictive proprioception. Micro-movements also allow automatic discounting of the input that makes the proprioception noisier and more random.

In short, through movement variability, understood not only as efferent motor output but also as kinesthetic re-afference in the context of stochastic processes, we offer a new unifying framework to (1) idiosyncratically quantify different levels of ASD in real time; (2) dynamically track real time and longitudinal performance in the context of decision-making; and (3) develop new personalized therapies that may exploit the sensory-motor capabilities of the autistic individual.

The micro-movement methodology does not depend on explicit instructions. It can track spontaneous behavioral variability and variability from deliberate behaviors. This means that individuals with ASD who are non-verbal or who may have difficulties acting on command will be able to benefit from personalized therapies that use their micro-movement statistics. This is important because many non-verbal individuals have already developed their own compensatory strategies undetectable by conventional methods. Our methods can detect and harness patterns from spontaneous behaviors that fall beneath the person's awareness and reflect some of those strategies.

We are at present using these methods to track longitudinal changes in spontaneous behavior before, during and after treatment of an FDA-approved clinical trial drug using insulin-like growth factor 1 in children with a diagnosis of autism of known etiology [specifically, in children with Phellan-McDermid syndrome (Phelan and Rogers, 1993)].

We have discovered that the continuous two-parameter Gamma family of probability distributions captures with high confidence level the velocity-dependent variability inherent to all human movements throughout typical and atypical development and adulthood. This developmental path is well-characterized with a scaling power-law relation that objectively captures a connection between patterns of micro-movements and performance in decision-making related to cognitive control. Points corresponding to neighboring individuals on the Gamma plane had similar micro-movement signatures and similar verbal capabilities. Each person's signatures shifted at a different rate as a function of stimulus and task context, potentially signaling different levels of behavioral flexibility unique to each individual. This result offers a new form of flexibility-based classification for neurodevelopmental—and neurodegenerative (Torres, 2013a)—disorders in general. This further enables flagging early on atypical signatures of kinesthetic re-afference. It also shows the tangible possibility of developing objective target therapies tailored to each person's predispositions, capabilities, and flexibility so badly needed in autism research and treatments.

### Too much noise: the corrupted kinesthetic re-afferece in ASD

As noted, the unveiled body micro-movements are also the immediate object of internal kinesthetic sensations as they shift signatures over time. Notice here that the problem may be at the motor output due, for example, to low muscle tone; it may be at the afferent synapses; it may be at the central level where commands are issued, etc. The point is that we can capture a read-out of the somatosensation of the person at the motor output—even without knowing the exact origins of the disturbances. We know that these stochastic fluctuations are being kinesthetically sensed by the system over time and impinged by external and internal influences. Thus, we can track those effects and efficiently, *in real time*, steer the system using adequate input. Our methodology objectively quantifies the dynamic sensation of re-afferent movements and thereby quantifies a form of proprioception. Our findings show a developmental trajectory wherein TD micro-movement proprioception undergoes maturation that results in specific probabilistic expectations. In the language of Bayesian statistics, such acquired “priors” allow the agent to make meaningful categorizations and sense unexpected internal and external disruptions through their own movements. We have also found that mature TD micro-movements can be separated into functional classes with different levels of intentionality. They operate at different time scales in their latencies to reach critical points (e.g., maxima) along the kinematic trajectory.

The measured experimental data shows that the path of micro-movement development is fundamentally different for the individuals diagnosed with ASD. Their hand movements appear to remain at the kinesthetic stage of TD 3–4 year old children and to some extent even regress as they veer off the typical developmental path. The data shows that regardless of age, the individuals with ASD do not acquire their own reliable statistical expectations from their behavioral variability (i.e., do not acquire reliable kinesthetic priors). Their sensory-motor signal is overpowered by noise and never diversifies.

### Linking kinesthetic re-afferent micro-movements, lack of social communication, and other behavioral symptoms in ASD

#### Lack of flexibility

Reliable kinesthetic priors are needed as anchors to measure new movement fluctuations; i.e., to establish an implicit embodiment of the statistics in the external signal. The empirical finding that ASD individuals do not acquire such expectations implies that they cannot discriminate different levels of functionality in their physical movements. A testable hypothesis is that it is unlikely that they would be able to discriminate different levels of functionalities in the movements of others, e.g., distinguish when a gesture is intentional from when the same gesture is spontaneous. Further, the lack of kinesthetic priors means that the individual with ASD lacks an implicit reference frame for new variations in different contexts. Thus, they cannot discriminate the variability of their own movements from contextual internal and external influences. Where the TD individual can purposefully sample and adapt to sensed changes, any attempt to diversify the input would amplify the noise and maximize uncertainty for the autistic individual. The reliance on sameness can be, at least in part, traced back to the lack of movement expectations or “kinesthetic priors.” These may also have downstream effects on perceptual and mental navigation. It forces the autistic system to rely on the concrete “here and now” of perceived body position and environment. All in all, we conjecture that these experimental findings may begin to unify and explain several of the key symptoms of ASD.

#### Sensory integration

The lack of reliable priors, the excess noise, and the lack of re-afferent diversity are likely to impede the integration of sensory inputs from different sensory modalities as there is no internalized sensory-motor frame of reference to organize the sensory integration. If so, this would restrict the autistic individual to rely on the modality that best works for his/her system, actively ignoring other modalities that would only amplify the sensory-motor noise and increase uncertainty. The sensory issues in ASD are multipronged. They often have an impact on their ability to sort information from single modalities at low-level processing. Likewise, for a hyper sensitive system, if everything is signal in certain sensory domains, how does that system filter out interfering signal (noise) from the relevant signal within a given sensory modality? Our new methods will allow further investigations of other potential underlying causes for the disruptions quantified here, including possible malfunctioning of the ANS and their relation to circadian rhythms regulating food-intake, sleep cycles, and gastro-intestinal functions. It may be possible to assess contributions of the peripheral noise-to-signal re-afferent feedback to the central regulation, coordination, and control of anticipatory sensory-motor integration.

#### Cortical and peripheral anchors

We propose the hypothesis that the typical development of “kinesthetic priors” is essential not only for anchoring kinesthetic sensing but also for the typical development of cortical sensorimotor circuits; circuits critical for flexible hierarchical action planning, shifts of attention, and establishing counterfactuals in symbolic problem solving. Experiencing kinesthetic re-afference as a stable percept serves as an abstract generalization that allows us to navigate and track action opportunities “off-line” without constant concurrent perceptual guidance. This hypothesis finds some support in the current hand micro-movement variations. The TD children younger than 4 years did not reliably show internalized priors—a result congruent with the maturation stages necessary to perform traditional theory of mind tasks (Baron-Cohen et al., 1985). The new methodology will enable further explorations into the nature of the shifts in stochastic signatures characterizing the morphing of noise into signal during flexible exchanges between intentional and spontaneous mode of behavior.

#### Accumulative social and communicative issues

It is very unlikely that individuals with ASD can make anticipatory decisions and estimate the consequences of their own impending actions in a timely fashion. This is suggested by the quantified random, noisy, and restrictive proprioception, prevailing across ages in the data set. Much less probable would be that they could apply fine-tuned discriminations to the actions and emotional facial micro-expressions of others during real time social interactions. Given the stochastic signatures of kinesthetic re-afference found here, it may be possible to investigate more precisely why it seems impossible for individuals with ASD to visually perceive intentional motions and weight their potential consequences, e.g., to “see” in real time the intentional movements in cartoons with geometric figures as shown in the classic Heider and Simmel experiment (Heider and Simmel, 1944).

It is our conjecture that the noisy, random, and restrictive proprioception of their own physical micro-movements impedes as well their visual perception of micro-movements in others during real time interactions. A congruent map between physical and visual perception of motion may be necessary for the correct interpretation of external movement patterns inherently present in social dynamics (Johnson et al., 2012a,b). Without basic kinesthetic re-afference in place it is very unlikely that flexible and timely discrimination between intentional and spontaneous gestures develops.

The proposed framework will permit us to deconstruct impairments in social interactions via the stochastic approach to assess in real time the non-stationary signals generated by our bodies and by the bodies of others in a social scene. These include speech, gestures, body poses as well as the velocity-dependent kinematics of the micro-expressions of the faces conveying emotional content. Micro-movements thus conceived as kinesthetic re-afference are present across all functional levels of the nervous system (in Figure 1B). If this input is noisy and unstable, the required map between visual and kinesthetic percepts of our own motions and those of others would be disrupted. This would make it impossible to co-adapt social interactions in real time and, in general to mentally navigate through social dynamics with successfully confirmed outcomes.

#### Coping and compensatory adaptive mechanisms

Lastly, we conjecture that the observed behavioral symptoms are dynamic byproducts of an individual coping with low-level corrupted signals. Similar to any other biological system, the autistic system may have found compensatory strategies to deal with corrupted re-afferent input and close the feedback loops to sustain a rudimentary form of (non-anticipatory) motor control. An intriguing result from this work is the effect of aging on the micro-movements of individuals with ASD. In the initial stages around 4–6 years of age the children with ASD studied were closer to the TD children of similar chronological age than to the children with ASD older than 8 years of age. Then their micro-movements' stochastic signatures not only veered-off the typical developmental path, it also reversed direction away from it. The empirical data suggests that as individuals with autism age, their micro-movements become even more random, noisier, and more restricted. Why this reversal? We hypothesize that this reversal is part of a dynamic *coping strategy* that ends up reinforcing a narrow bandwidth of sensory input embedded in unreliable re-afferent information from their physical actions. If sensory-motor integration fails and the system cannot spontaneously form proper maps of the body in space and time and filter relevant perceptions, then exploration can only bring more uncertainty.

TD individuals can anchor their explorations in implicit predictable priors that allow variations to become informative signals. They can then adaptively reshape these priors on demand. For individuals with nearly “memoryless” statistics and little implicit sense of their own bodies—every variation becomes noise. Thus, the intense desire for sameness—and to some extent the avoidance of social interactions—can be seen as active attempts to limit uncertainty (noise) in an already noisy and non-diversified input. Some repetitive motions can be understood as part of a search for current verification of body position in space, which would help not only the impaired implicit body map but also could be used to “keep out” confusing and perplexing noise from the broader environment. Restricted interests can be seen as higher-level attempts to create predictable environmental pockets where expectations hold. This yields a memory/world-based predictability and a sense of safety whereby nearly no adjustment is needed for successful actions.

In accordance with the idea of successful coping, we saw that individuals with ASD often had a high accuracy in the match-to-sample decision, in spite of their corrupted proprioception. This suggested that they rely on an alternative strategy. Where the embodiment of the statistics of the external physical input is likely to underlie the fast and accurate decisions in TD individuals, the improvements in decision-making accuracy in the ASD individuals must depend on alternative means such as the concrete physical input (e.g., the visual feedback present throughout the decision period).

Future research and therapies will need to be more alert to disentangle the atypical behavioral phenotypes of people with ASD into original impairments and/or active successful coping behaviors. Whereas, our actions rely on anticipatory and mentally controlled and regulated physical motor outputs rooted in highly expected variability, the ASD individual must rely on the concrete here and now with minimum likelihood for anticipation and mental control and regulation of the efferent motor output.

One could argue that the way forward will involve an analysis of idiosyncratic micro-movement challenges and an individualized treatment approach that exploits not only whatever movements a particular individual on the spectrum can manage but also whatever adaptations his/her different neurology and experience have afforded him/her. Here individuals with autism could play an active role in helping us figure out why they have been able to come so far with the movement challenges they obviously have (Savarese, 2013).

### Future steps

While we provide here a new framework and a set of objective metrics to dynamically study both typical and autistic traits, we are still far from explaining the causes of these atypical micro-movements to get closer to the true underlying causes of autism. Our results suggest that there is a lack of spontaneous autonomy in the autistic system that impedes adaptive and co-adaptive volitional control. These may be largely contributed by corrupted afferent peripheral information, including input from the autonomic and somatic nervous systems of which we specifically tackled hand movement proprioception here. Our work highlights that autism is a systemic neurodevelopmental disorder with concrete, measurable physical bases. Autism should not be exclusively portrayed as a psychological, abstract cognitive/social problem of a *“disembodied”* brain. That would be merely a static snapshot of a person whose sensory-motor systems are clearly evolving and changing in adaptive and compensatory ways.

The metrics and framework offered here provide a complementary and new way to unify brain and body interactions rather dynamically. We can now study the dynamic contributions from peripheral afferents in tandem with centrally sent signals and aim at evoking and maintaining in the autistic individual better regulation and anticipatory control of the efferent output signals. We need to exploit the capabilities inherent in individuals coping with and adapting to sensory-motor problems of genetic and/or epigenetic origins. By connecting and being able to measure central and peripheral contributions objectively we can redefine autism in relation to phylogenetic constraints involving synapses and networks at all levels of the nervous systems (not just at the cortical level). More importantly, we can find new avenues for personalized target treatments—*even before we get at the causes of autism*.

It is clear, nonetheless, that we need to look beyond the limb movements explored in this current study. In the future we need to explore the possibility that the noisy and narrowed-bandwidth proprioception of the limbs and hand motions may extend to all functional levels of micro-movements including those embedded in speech and facial micro-expressions. Sensory-motor orofacial nerves are phylogenetically and anatomically different from those of the limbs. We hypothesize different developmental timelines and cognitive effects for orofacial proprioception than those which we have found for hand and arm-based movements. This is a testable hypothesis under the present framework. Further, conceiving motion across multiple functional levels as a *change of position over time*, we can apply the current statistical metrics to objectively measure all sensory levels of biological beings in real time (Figure 2). Thus, we propose to further use the present methods to understand autism above and beyond perceptible differences with TD controls.

Despite the systemic problems identified with movement-based proprioceptive information, a positive result emerged from the decision-making experiment. Most participants with ASD experienced a shift of their micro-movements as a function of changes in match-to-sample stimuli. In several cases and for specific stimulus types this shift was toward the predictive limits of the Gamma plane. This implies that, at least transiently, changes in sensory input can (1) be detected by the autistic systems and (2) help anchor movements in such a way as to make pointing motions more predictable. Even though internal feedback is corrupted, reliance on the concrete physical reality allows external anchoring to close the feedback loops and support adaptive exploration: i.e., a form of sensory substitution that we can link to patterns of spontaneous micro-movements. In another paper of this Research Topic (by Torres et al.) we show how in a matter of seconds computerized behavioral interventions requiring no instructions can lead non-verbal children with ASD toward the spontaneous self-discovery of a goal and the autonomous and more anticipatory control of motions: They attain a reward and sustain it under a form of *acquired* adaptive volitional control. The participants in that study retained their shifts weeks later. Even without practice their micro-movements shifted toward anticipatory, intentional features. We were able to automatically and objectively track these changes longitudinally using the current framework and metrics. In this sense we already know that in several of these children there were long-term gains in predictability and reliability of their actions, not just transient changes within a given session.

In the present work we also registered negative gains toward the Exponential range of the Gamma plane as the individual adapted to the new task context. We can test *selectively* in real time which sensory modality may better guide each person toward predictive or preferred performance. In other words, we can automatically detect which form of sensory input most likely accelerates the learning progression with minimum resistance by the child's sensory-motor systems. This would be the sensory input with the largest rate of change toward higher predictability (highest value of the shape parameter toward the Gaussian limit of the Gamma plane) and highest reliability (the lowest dispersion which corresponds to the lowest Fano Factor given by the variance to mean ratio). We can reinforce that source of sensory guidance and discount the sensory modality that makes the kinesthetic percept noisier and more random.

To the best of our knowledge this is a new way to objectively and dynamically track in real time the shifts in stochastic signatures of the non-stationary statistics of the continuous flow of natural behavior. We have developed a methodology that permits very precise and automatic assessment of the form of guidance that most rapidly improves re-afferent kinesthetic input and accelerates learning. This in turn leads to an enhanced volitional control of the child over his/her motions and the development of better autonomy over the connection between his/her intentions and actions. This is the first inclusive methodology that harnesses the sensory-motor capabilities and the adaptive learning predispositions that are already present in the individual with ASD.

Our work differs fundamentally from current behavioral training techniques which rely on commands and a priori selected stimuli. Our next immediate goal is to design new metrics that can tell us exactly the path of least resistance [in a very precise physical sense (Lanczos, 1966; Feynman et al., 2006)]: the path which accelerates learning and moves the child's kinesthetic re-afference away from the rim of maximum uncertainty. Overall, we found that each individual in the spectrum is *unique* and learns at a unique rate, a result that we would have missed had we assumed homogeneity a priori, formed groups accordingly, and assumed a priori an underlying probability distribution common to the entire ASD cohort.

Although we have used here human participants to illustrate the use of the new framework, micro-movements are inherent to any biological organism with sensory transducers, which autonomously moves to survive and reproduce. Our framework can also be applied to objectively analyze behavioral phenotyping assays in animal models of autism (and other spectral disorders) to evaluate important contemporary emerging theories that will guide our quest for the causes of ASD and for clinical treatments (Markram and Markram, 2010; Silverman et al., 2010).

In summary, we have shown that studying the statistics of micro-movements' variability provides a powerful tool to build a new generation of objective diagnostic assessments of ASD. These include new metrics to assess the long-term flexibility and plasticity of sensory-motor systems in the face of compensatory adaptive mechanisms self-discovered by the person with ASD on a short-term basis. The new methodology will enable the development of new personalized interventions tailored to the individual's inherent capabilities. The individual with ASD does not develop by default the predictability of micro-movements that allows for anticipatory, adaptive, and explorative behavior. However, applying our new methods has allowed us to uncover new ways to evoke real time transient changes toward predictive behaviors with long-lasting effects retained weeks later (Torres, 2013a,b in this issue).

We have quantified ways to evoke shifts toward predictive statistical movement regimes as well as changes toward faster and more accurate decisions. This, despite the quantification of movement sensing that appears to be overpowered by noise and lacking diversification. With this new methodology we can now explore the heterogeneity of ASD and enhance cognitive learning predispositions inherently present in each child. We studied not only goal-directed movements but also spontaneous behavioral variability present in incidental motion segments (largely beneath the person's awareness). Such motion segments pursued no concrete goals. They provide new means to objectively quantify changes in a type of cognitive learning that occurs without explicit instructions and largely without concrete purpose. Future work will extend this quantification to other automatic and autonomic levels across other populations of neurodevelopmental spectral disorders of known and of unknown etiology.

### Conflict of interest statement

The authors declare that the research was conducted in the absence of any commercial or financial relationships that could be construed as a potential conflict of interest.

## Appendix

**Table A1:** **Scores from clinical assessments of the participants with ASD**.

**Self-emerging**	**Code**	**Gender**	**Age (yrs)**	**Stanford-Binet**			**ADOS scores**				**GARS scores**			
**Cluster**				**NVIQ**	**VIQ**	**FSIQ**	**Stereo**	**Com**	**Soc**	**Com + Soc**	**Stereo SS**	**Com SS**	**Soc SS**	**Autism index**
1	01	M	4.3	42	43	40	4	8	13	21	N/A	N/A	N/A	N/A
1	02	F	5.9	44	51	45	2	4	13	17	N/A	N/A	N/A	N/A
0	03	M	6.0	N/A	N/A	100	N/A	N/A	N/A	N/A	N/A	N/A	N/A	N/A
1	04	M	6.3	N/A	N/A	50	N/A	N/A	N/A	N/A	N/A	N/A	N/A	N/A
1	05	M	7.6	50	46	45	3	6	11	17	N/A	N/A	N/A	N/A
1	06	F	7.8	42	43	40	4	7	12	19	N/A	N/A	N/A	N/A
2	07	M	7.8	42	43	40	2	6	14	20	N/A	N/A	N/A	N/A
2	08	M	9.0	42	44	40	1	5	10	15	N/A	N/A	N/A	N/A
2	09	M	9.9	42	43	40	4	5	8	13	N/A	N/A	N/A	N/A
3	10	M	10	N/A	N/A	107	3	3	9	12	N/A	N/A	N/A	N/A
2	11	M	10.3	42	43	40	3	4	10	14	N/A	N/A	N/A	N/A
0	12	M	11.5	100	82	90	7	5	6	11	N/A	N/A	N/A	N/A
2	13	F	11.5	50	43	44	N/A	N/A	N/A	N/A	N/A	N/A	N/A	N/A
2	14	M	11.7	42	43	40	5	8	10	18	N/A	N/A	N/A	N/A
2	15	M	11.7	43	43	40	N/A	N/A	N/A	N/A	N/A	N/A	N/A	N/A
3	16	M	12	N/A	N/A	67	4	5	13	18	N/A	N/A	N/A	N/A
3	17	F	12	N/A	N/A	60	4	8	10	18	N/A	N/A	N/A	N/A
3	18	M	12	N/A	N/A	95	2	5	8	13	N/A	N/A	N/A	N/A
3	19	M	12	N/A	N/A	95	1	5	7	12	N/A	N/A	N/A	N/A
3	20	M	13	N/A	N/A	89	2	3	7	10	N/A	N/A	N/A	N/A
2	21	M	13.8	42	43	40	N/A	N/A	N/A	N/A	N/A	N/A	N/A	N/A
3	22	M	14	N/A	N/A	74	3	9	10	19	N/A	N/A	N/A	N/A
2	23	F	14.3	50	43	44	N/A	N/A	N/A	N/A	8	11	9	124
3	24	F	15	N/A	N/A	52	2	6	11	17	N/A	N/A	N/A	N/A
3	25	F	15	N/A	N/A	77	N/A	N/A	N/A	N/A	N/A	N/A	N/A	N/A
3	26	F	15	N/A	N/A	71	6	5	7	12	N/A	N/A	N/A	N/A
3	27	M	15	N/A	N/A	56	3	4	10	14	N/A	N/A	N/A	N/A
2	28	F	15.8	42	43	40	N/A	N/A	N/A	N/A	13	10	11	109
3	29	M	16	N/A	N/A	100	N/A	N/A	N/A	N/A	N/A	N/A	N/A	N/A
3	30	F	16	N/A	N/A	81	2	7	9	16	N/A	N/A	N/A	N/A
3	31	M	18	N/A	N/A	101	2	4	6	10	N/A	N/A	N/A	N/A
3	32	M	18	N/A	N/A	96	4	4	8	12	N/A	N/A	N/A	N/A
3	33	M	18	N/A	N/A	76	1	5	7	12	N/A	N/A	N/A	N/A
0	34	M	25	N/A	N/A	99	6	3	7	10	N/A	N/A	N/A	N/A

First column identifies the self-emerging cluster number in Figure 8B (1 magenta, 2 maize, 3 black, 0 is for outliers). Stanford-Binet 5th edition was used to assess intelligence of each participant with ASD (Roid, 2003). A score of 100 is the norm and a departure by 15 points indicates one standard deviation above or below typical intelligence. NVIQ is a measure of non-verbal IQ. VIQ is a measure of Verbal IQ. FSIQ is the sum of verbal and non-verbal intelligence scores converted to a standardized score. Autism Diagnostic Observational Scale (ADOS) (Lord et al., 2000; Gotham et al., 2009) is a standard assessment tool used by clinicians as a basis for the ASD diagnosis. Module 1 of the ADOS was used for the young, non-verbal students. Module 3 was used for the adolescent students with conversation ability. Stereo is a measure of stereotyped behaviors were a higher score indicates more stereotyped behaviors; however without cutoff for a ASD diagnosis. Com is the total Communication score, where 4 is the cutoff for Autism and 2 the cutoff for Autism Spectrum. Soc is the total Reciprocal Social Interaction Score, where 4 is the cutoff for Autism, and 2 the cutoff for Autism Spectrum. Com + Soc is the combined Communication and Social Interaction score, with a score of 12 being the Autism cutoff, and 7 the Autism spectrum cutoff. Because of their age and extremely limited verbal ability, 2 children could not be given the ADOS. Therefore, the GARS 2 (Gilliam Autism Rating Scale – Second edition) (Gilliam, 2006) was used to assess these individuals. Stereo SS is the standardized score of stereotyped behaviors. Com SS is the standardized score of Communication. Social SS is the standardized score of Social Interaction. The Autism Index is the sum of standard scores, converted to normed index score.

**Table A2:** **Information from TD participants**.

**Average speed cluster**	**Participant**	**Gender**	**Norm speed typical cluster**
4	1	M	1
4	2	M	1
4	3	F	1
4	4	F	1
4	5	M	1
4	6	M	1
4	7	F	2
5	8	F	2
5	9	F	2
5	10	M	2
5	11	M	2
5	12	F	2
5	13	M	2
6	14	F	3
6	15	F	3
6	16	M	3
6	17	M	3
6	18	F	3
6	19	M	3
6	20	F	3
6	21	F	3
6	22	M	3

TD children were recruited from the “small wonders” class at the Rutgers Douglass Developmental Disability Center (DDDC). This is a group of typically developing children who go to the DDDC for pre-school because one of their parents/caregivers works at the DDDC. These students are an excellent control group to the ASD target group, because all children share the same learning environment. They are represented by clusters 4 (green) and 5 (blue). Cluster 6 (red) consists of college-level participants who served as controls for the high-functioning ASD group (Cluster 3, black).

**Table A3:** **Information from College-Level TD participants**.

**College level TD participants**		
**ID**	**Gender**	**Age (yrs)**
1	M	19
2	M	20
3	M	20
4	F	20
5	M	20
6	F	21
7	M	21
8	M	21
9	M	21
10	F	21
11	F	22
12	F	22
13	F	22
14	M	23
15	M	23
16	M	23
17	F	23
18	F	23
19	F	24
20	F	25
21	M	60
22	M	61

**Table A4:** **Population Gamma estimated values and 95% confidence intervals for each group across all trials for the time-normalized path length**.

**Group**	**[Min, Max] median (m)**	**Gamma fit shape scale**	**Confidence intervals 95%**
TD1 (green)	[0.09, 0.1495]	1.344	[1.2915, 1.3995]
IQ ≈100, Age 3–4	[0.05, 0.1481]	0.015	[0.0148, 0.0163]
	0.0525	1.427	[1.3713, 1.4869]
	0.0530	0.014	[0.0139, 0.0153]
TD2 (blue)	[0.0357, 0.3246]	9.5752	[8.7742, 10.4493]
IQ ≈100, Age 4–5	[0.0430, 0.3556]	0.0115	[0.0105, 0.0126]
		9.7816	[8.9779, 10.6571]
		0.0118	[0.0108, 0.0129]
	0.1061		
	0.1199		
TD3 (red)	[0.1974, 0.3962]	88.9370	[77.3933, 102.2025]
IQ >100, Age 21–30	[0.1476, 0.4096]	0.0032	[0.0028, 0.0037]
		111.2034	[96.7815, 127.7744]
		0.0026	[0.0023, 0.0030]
	0.2856		
	0.2958		
ASD1 (magenta)	[0.089, 0.3172]	3.3356	[3.3750, 3.8621]
IQ <50, Age 4–8	[0.0104, 0.3586]	0.0246	[0.0208, 0.0240]
		3.6286	[3.5830, 4.1003]
		0.0249	[0.0217, 0.0251]
	0.0896		
	0.0924		
ASD2 (maize)	[0.070, 0.9880]	1.9132	[1.8578, 1.9701]
IQ <50, Age 8–16	[0.076, 0.9905]	0.0676	[0.0653, 0.0699]
		1.9660	[1.9090, 2.0247]
		0.0735	[0.0711, 0.0760]
	0.1012		
	0.1355		
ASD3 (black)	[0.0541, 2.9901]	2.3684	[2.3058, 2.4328]
IQ <50, Age 10–25	[0.0485, 2.9819]	0.1867	[0.1812, 0.1924]
		2.3458	[2.2839, 2.4094]
		0.2092	[0.2030, 0.2155]
	0.3474		
	0.3878		

Analyses of the average speed variability. Values are from each self-emerging cluster showing 95% confidence regions for estimated moments and Gamma parameters. First line in each row is from the forward segment communicating the decision. Second row is from the goal-less retractions. In terms of the overall scatters in Figures 8B,C, for the forward movements of the TD participants their age correlated positively with the a-shape parameter (r = 0.879, p < 0.01, 2-tailed), whereas the b-scale parameter correlated negatively with age (r = −0.942 p < 0.01, 2-tailed.) These correlations maintained the signs in the retracting movements (age vs. shape r = 0.906, p < 0.01 and age vs. scale r = −0.893, p < 0.01.) In the participants with ASD the correlations were significant, but the overall pattern of them differed for age (and IQ.) The reported IQ was examined in relation to the stochastic (a,b) signatures. With the exclusion of the 3 outliers (see discussion in results) the correlation coefficients were: In the forward motions the a-shape parameter correlated positively with IQ (r = 0.415, p < 0.05, 2 tailed) but was not correlated with age. The b-scale parameter correlated positively with age (r = 0.642, p < 0.05, 2 tailed) but was not correlated with IQ. These patterns were maintained in the retracting motions as well (a-shape, r = 0.412, p < 0.05, 2-tailed; b-scale, r = 0.582, p < 0.01, 2-tailed.) Inclusion of outliers maintained the patterns of significance but lowered the coefficients (forward, a-shape vs. IQ r = 0.440, p < 0.001—but no age correlation, forward, b-scale vs. age, r = 0.380, p < 0.05—but no IQ correlation; retraction, a-shape vs. IQ, r = 0.406, p < 0.05, and b-scale vs. age, r = 0.395, p < 0.05).
